# Mucofilm: a nexus for phage-microbiome interactions in gut ecology

**DOI:** 10.1128/aem.01269-25

**Published:** 2025-10-14

**Authors:** Ania Stenberg Mortensen, Dennis Sandris Nielsen, Henriette Lyng Røder, Torben Sølbeck Rasmussen

**Affiliations:** 1 Section of Food Microbiology, Gut Health and Fermentation, Department of Food Science, University of Cophenhagen 545332 , Frederiksberg, Denmark; Michigan State University, East Lansing, Michigan, USA

**Keywords:** human gut microbiome, gut ecology, biofilm, bacteriophages, bacteriophage-bacterial interactions, resilience, spatial distribution, reservoir

## Abstract

The human gut is a dynamic ecosystem where bacteriophages (phages) and bacteria interact within a complex mucosal environment. Here, we introduce the concept of mucofilm, a hybrid matrix composed of mucus produced by host cells and bacterial biofilm, as a unified ecological niche in the gut while also recognizing its relevance to other mucus-covered surfaces. Traditionally treated as separate entities, mucus and biofilm are in fact deeply interwoven, forming a complex environment that shapes microbial interactions, phage dynamics, and host responses. We question whether traditional knowledge about phage-biofilm interactions is transferable to mucofilm, and we therefore believe that recognizing mucofilm as a distinct structure is essential for understanding how phages interact with the gut microbiome, influencing microbial resilience, diversity, and immune modulation. This commentary challenges conventional compartmentalization and highlights the need to consider mucofilm as a single, integrated system when designing microbiome and phage-related studies. By doing so, we can better predict microbial behavior and improve therapeutic strategies targeting gut-associated diseases.

## COMMENTARY

Understanding the true nature of *in vivo* biofilms is essential for advancing both basic and applied microbiology. All *in vivo* biofilms, except for oral plaque, are embedded in human-derived macromolecules (e.g., mucin) (1). This stands in contrast to the classical definition of a biofilm, such as the one provided by the International Union of Pure and Applied Chemistry (IUPAC), which defines a biofilm as *“*aggregates of microorganisms in which cells are frequently embedded in a self-produced matrix of extracellular polymeric substances (EPS) that are adherent to each other and/or a surface” (2, 3). One could therefore argue that *in vivo* biofilms represent a distinct entity, as the combination of host-derived macromolecules like mucin polymers and microbial EPS gives rise to a hybrid matrix not captured by the traditional definition. Without a common terminology and definition, progress risks being fragmented; conversely, reaching agreement would enable the field to consolidate and build on current knowledge more efficiently.

## MUCOFILM: A CENTRAL NEXUS IN GUT MICROBIOLOGY

The human gut is a highly complex ecosystem where bacteria primarily form multispecies communities with intricate inter-kingdom symbiosis. The stability of these communities is tightly linked to host health and disease, and even minor disruptions may significantly impact homeostasis, and therefore, it is essential to understand how and when these communities are impacted (4). An important player shaping these microbial networks is phages. As natural regulators of bacterial communities, phages influence microbial interactions in all habitable ecosystems (5). By exploring how phages influence these bacterial interactions, we may improve our ability to predict and control the development of these communities.

Phages are renowned for their ability to selectively target bacterial cells (6). This specificity makes phages a promising tool for targeting pathogenic bacteria that form biofilms, offering a potential complement to antibiotic therapies (7). Given their ubiquity in bacterial habitats, including the human gut, phages contribute to the shaping and stabilization of microbial communities, actively influencing bacterial adaptations and ecosystem structures (8, 9). However, the role of phages is not isolated—phage-bacteria interactions are influenced by their surroundings, and in the human gut, this means a highly dynamic environment shaped by both biotic and abiotic factors.

The gut environment—characterized by continuous mucus secretion from goblet cells, intricate biochemical signaling, and various abiotic factors such as temperature, pH, tissue architecture, and peristalsis—creates diverse and dynamic microbial niches (10). It is the interplay of these elements that exerts selective pressure on bacteria, driving them to adapt in order to survive. One of the most widespread adaptive strategies is biofilm formation, which enables bacteria to colonize within these niches by embedding themselves in a self-produced extracellular matrix (6, 11). When this survival strategy is employed, bacterial organization, resilience, and interactions with both microbes and host cells are enhanced (6). This structural adaptation not only supports microbial diversity but also directly influences phage diversity, mobility, and infection dynamics within the gut ecosystem (12). These biofilms have been found to be integral to the human gut (11, 13).

Through millennia of co-evolution, the human host and gut microbiome have formed an intricate, interwoven system in which biofilm-forming bacteria and mucin proteins work together to maintain a delicate balance (12, 14). Electron microscopy has revealed bacterial colonies characteristic of biofilms in the human gut, particularly in the mucus lining along the gut epithelium (13). The mucus layer, composed of host-derived mucins, shields epithelial layers exposed to the external environment in several locations in the body, from the eyes, lungs, and oral cavity to the urogenital, cervicovaginal, and gastrointestinal tracts (1, 15–18). Defining how the mucus layer influences *in vivo* biofilm formation remains essential.

**Fig 1:**
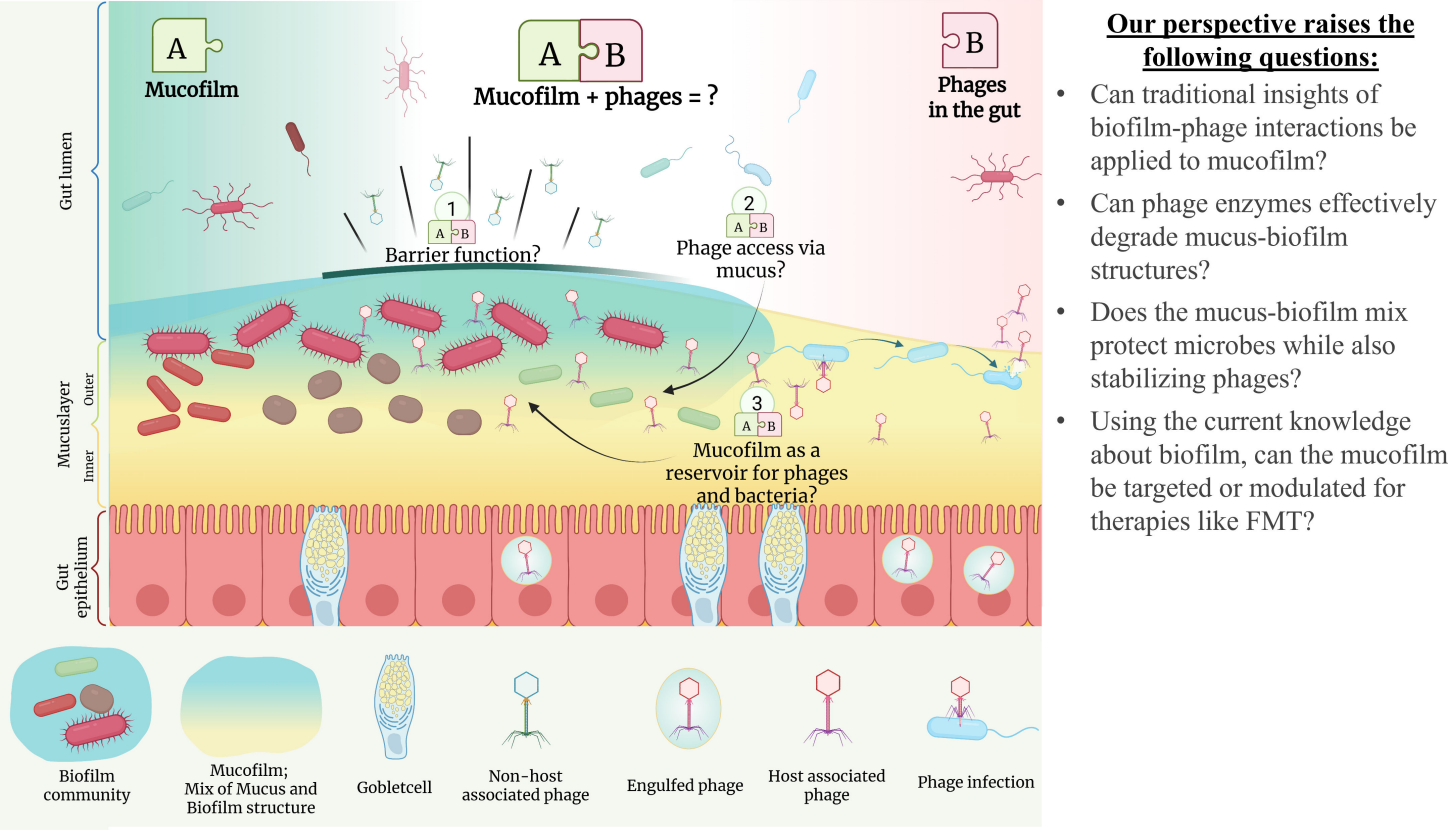
Phages in the mucofilm of the gut epithelium. The mucofilm protects the bacteria from environmental stressors, such as changes in pH, salinity, and the introduction of new phages. It might be regulated by the phages that adhere and proliferate in the mucosal layer, acting as a secondary non-host-derived immune system against pathogenic bacteria. To fully understand the gut ecosystem, we should combine both research in mucofilm and gut-associated phages as they are two parts of the same system. Here, we consider the attributes of mucofilm and its effect on phage properties (1-3).

These mucus-rich surfaces also act as reservoirs for commensal and potentially biofilm-forming bacteria, allowing microbial communities to persist (10, 19). The gastrointestinal tract contains one of the most complex ecosystems localized within the mucosal layer that varies in characteristics along the length of the intestine (20). In the small intestine, the mucus is relatively thin (duodenum ~170 µm, jejunum ~125 µm, ileum ~480 µm), whereas the colon harbors a thick (~830 µm) double-layered mucus structure (10, 19, 21). The two-layered nature of the colon mucus provides a dense, inner layer which is generally considered free from bacteria, and an easier-to-colonize outer layer serving as a habitat, reservoir, and partial energy source for commensal bacteria (10, 19). The properties of the mucosal layer and gut-associated biofilms likely lead to their intermingling, creating a hybrid matrix that enhances bacterial persistence and shields microbes from environmental stressors like antibiotics, host-derived antimicrobial peptides, and other perturbations (10, 11). We propose the term “mucofilm” to describe the unique combination of biofilm-forming bacteria and host-derived mucus, and we question whether traditional knowledge about phage-biofilm interactions is transferable to mucofilm. Despite progress in the field, much remains unknown about the gut ecosystem—particularly interactions between phages and mucofilm communities, which limit insight and therapeutic development. This comment presents new perspectives and introduces a conceptual framework (Fig. 1) to better define and explore this complex interaction and guide future research.

## ROLES OF PHAGES IN GUT ECOLOGY AND INTERACTIONS WITH HOST CELLS

The integral role of mucofilm in shaping microbial communities underscores the crucial need to examine how phages and bacteria interact within this complex environment. Fecal virome transplantation (FVT) has demonstrated that phages play a key role in treating recurrent *Clostridioides difficile* infections and preventing necrotizing enterocolitis in preterm piglets—emphasizing the therapeutic potential of phage-based interventions for gut-associated diseases (22). Phages, as bacterial predators, influence bacterial stability in the gut through their ability to drive bacterial diversity and community shifts (9, 23, 24) and transfer functional genes (8, 11). Beyond bacterial modulation, phages are also able to bind to mucin proteins in the gut, forming a secondary, non-host-derived immune defense that helps prevent bacterial invasion in areas with high phage density (9, 25). Growing evidence is showing that, like eukaryotic viruses, phages can be actively internalized by host cells, generating cytokine release that recruits adaptive immune cells (9), among others through their persistence within mucosal epithelial barriers (26). These mechanisms may impact host immunity (9).

## MUCOFILM DYNAMICS AND PHAGE INTERACTIONS

Mucofilm, a mixture of biofilm components and mucus, establishes a highly complex microbial environment. Within this matrix, phages face both opportunities and challenges (Fig. 1)—particularly if the biofilm is interwoven with mucin, a highly glycosylated protein abundant in the intestinal mucus layer. The glycoproteins in the mucus layer compound these challenges, forming an additional barrier for the phages to penetrate. However, this same glycosylated structure may also support phage persistence: By adhering to mucin via, e.g., Ig-like domains, phage populations could engraft within the mucofilm, effectively increasing their local concentration and potentially amplifying their impact. Beyond these interactions, the colonic mucus layer itself is a responsive biomaterial, with its structure dynamically modulated by microbes as they degrade dietary polymers such as fibers—a factor that must be considered in future studies combining dietary components and gut commensals (27). This dual role of the mucofilm, both as a barrier and as a reservoir, defines it as a key player in microbial dynamics and underscores the need to recognize it as a distinct ecological setting. Walsh et al. (2024) highlighted how this intermingling of host mucus and microbial biofilms alters matrix properties (28). Using an *in vitro* model of endotracheal tube biofilms with a synthetic ventilated airway mucus medium, they could observe both higher minimum inhibitory concentrations (MICs) and minimum biofilm eradication concentrations (MBECs) for biofilms of *Pseudomonas aeruginosa*, *Klebsiella pneumoniae,* and *Candida albicans,* compared with use of non-mucus-enriched media (LB-medium) (28). Furthermore, they saw that the presence of mucus changed both the matrix composition as well as the structure and susceptibility to matrix degrading enzymes for *P. aeruginosa*, *K. pneumoniae,* and *C. albicans* biofilms (28).

Although the high bacterial density in biofilms theoretically provides an ideal setting for phage replication and transmission, biofilm architecture also presents formidable obstacles for phages (6). Many phages possess depolymerases, enzymes that degrade capsular polysaccharides or exopolysaccharides, which facilitate access to bacterial surfaces through biofilm degradation. Other phage-encoded enzymes, such as endolysins, may further degrade the biofilm matrix, granting entry to well-guarded bacterial cells (6). However, does this mechanism also apply to mucofilm? Furthermore, dense extracellular matrices and their composition, spatially structured bacterial communities, collectively reduce phage infectivity, limiting their ability to spread (7, 11, 12, 29).

In addition to these physical barriers, secreted biofilm molecules can act as decoys, further impeding phage access by impairing diffusion through the matrix (6). Low bacterial metabolic activity may further hinder phage propagation, while the presence of phage-resistant phenotypes in the biofilm can allow subsets of bacteria to persist despite phage pressure (6).

Within the mucofilm, these interactions take on even greater evolutionary significance. The interplay between biofilm-forming bacteria, non-biofilm-forming commensals, and phages creates a dynamic predator-prey relationship, where phage predation exerts selective pressure on bacterial populations. The mucofilm may amplify these pressures, accelerate bacterial diversification, and shape microbial community composition (12). By understanding these evolutionary forces, we can gain deeper insight into microbial dynamics and explore novel therapeutic approaches that aim to manipulate bacterial and phage populations in the gut.

Ultimately, the efficacy of phages in navigating the mucofilm likely depends on their enzymatic capabilities, the charge of phage-associated proteins, their ability to interact with human epithelial cells, and the structural properties of the biofilm itself.

## FROM CONCEPT TO MODEL: EXPERIMENTAL ROUTES TO STUDY MUCOFILM

Capturing the complexity of the mucofilm ecosystem will require methods that combine mucus with physiologically relevant conditions—bridging anaerobic gut niches and aerobic, mucus-rich surfaces, such as those of the lungs. A fundamental challenge in modeling gut conditions is that most gut microbes require anaerobic growth conditions, whereas epithelial cells depend on oxygen (15). McCright et al. developed a gut model with physiologically relevant mucus properties by co-culturing Caco-2 enterocyte-like cells with HT29-MTX goblet-like cells (15). After 21 days, the Caco-2 cells matured into more representative gastrointestinal epithelial cells, and in combination with HT29-MTX cells, the system recapitulated key aspects of intestinal mucus physiology (15). While this study demonstrates the promise of *in vitro* models for mimicking *in vivo* conditions, it also highlights the persistent difficulty of reconciling aerobic requirements of epithelial cells with the anaerobic environment required by the gut microbiome. However, concepts like intestine-on-a-chip developed by Jalili-Firoozinezhad et al. could help overcome these challenges (30). This platform employs Caco-2 intestinal epithelial cells to mimic the epithelial layer but uniquely delivers oxygen directly to the eukaryotic cells while maintaining anaerobic conditions in the flowing media above them (30). Another promising approach is the MINERVA system, introduced by Sardelli et al., which supports the growth of intestinal microbiota under dynamic conditions within a 3D mucus model (31).

## CONCLUSIONS

Treating the mucus layer and the biofilm matrix as separate entities risks oversimplifying this complex environment. Mucofilm, an amalgamation of mucus and biofilm, represents a vital intersection between bacterial communities, phages, and human health. It highlights the intricate roles of biofilm-forming bacteria and host-derived mucus in shaping the gut ecosystem. Acting as both a protective shield and a dynamic environment, the mucofilm fosters bacterial resilience while challenging the efficacy of the phage. This unique matrix also holds therapeutic potential, particularly in the targeting of pathogenic biofilms. Understanding biofilm and gut mucus as one entity (mucofilm) will be crucial for advancing microbiome research, phage-based therapies, and strategies to enhance gut health and immune function.
